# Immunocompetent host and mortality-related coccidioidomycosis: A case report and narrative review

**DOI:** 10.1016/j.mmcr.2026.100766

**Published:** 2026-01-21

**Authors:** Efrén Rafael Ríos-Burgueño, Luis Antonio Ochoa-Ramírez, Ismael Velarde-Rodríguez, Juan Manuel Ramírez-Sánchez, Víctor Michael Salinas-Torres, Jesús Salvador Velarde-Félix

**Affiliations:** a Hospital General de Culiacán “Dr. Bernardo J. Gastélum”, IMSS Bienestar, Sinaloa, Mexico; b Universidad Autónoma de Sinaloa, Sinaloa, Mexico

**Keywords:** Coccidioidomycosis, Fluorinated steroids, Immunocompetence, Mortality

## Abstract

Coccidioidomycosis, caused by *Coccidioides* infection, is typically subclinical and self-limited in immunocompetent patients. Occupational soil exposure, age ≥55 years, pregnancy, comorbidities, or immunosuppression states are common contributors to disease progression and potential mortality. This report describes a 34-year-old woman from Northwest Mexico, without evident risk factors, who presented chest pain and odynophagia as initial clinical symptoms and died at the 19th day of progressive clinical evolution consequence of pulmonary coccidioidomycosis. Findings in this report, along with those reviewed from the literature, highlight potential disparities that may be helpful in limited clinical settings, so eventual mortality-related coccidioidomycosis can be prevented.

## 1 Introduction

Coccidioidomycosis is a disease caused by fungi of the genus *Coccidioides*, characterized by heterogeneous clinical manifestations, ranging from silent to pulmonary and disseminated forms, which can lead to multisystem organ failure and demise [1].

The clinical presentation and outcomes of coccidioidomycosis may be influenced by typical risk factors such as host's ethnicity, pregnancy, advanced age, comorbidities, occupational hazards, and immunological status [2].

Particularly, primary immunodeficiencies have been deemed as less common risk factors, but can have a strong negative impact on health, promoting susceptibility to *Coccidioides* infection [3]. Of note, coccidioidomycosis is typically mild or subclinical and self-limited in immunocompetent patients.

This report describes an apparently healthy young woman who died on the 19th day of clinical deterioration as a result of pulmonary coccidioidomycosis. In addition, a narrative review based on immunocompetent patients without risk factors facing mortality-related coccidioidomycosis was performed.

## 2 Case report

The patient was a 34-year-old, previously healthy, single woman, born and resident of Culiacan, Sinaloa, Mexico, where she worked as a taxi driver. Her medical history showed no clinical evidence of primary immunodeficiency, thyroid, or autoimmune disorders, diabetes mellitus, hypertension, cancer, nor history of recurrent respiratory or gastrointestinal infections; whereas her family history was also unremarkable. Furthermore, an obstetrical history of pregnancies as well as drug addiction, alcoholism, smoking, previous hospitalizations, household overcrowding, and frequent national or international travel history were denied.

The patient presented with chest pain and odynophagia as initial clinical symptoms. A primary care physician prescribed an empirical treatment based on ceftriaxone (1 g daily for 3 days) and dexamethasone (8 mg daily for 6 days), both intramuscular. In the following 24 hours, the patient experienced dyspnea and dry cough, then intravenous and oral levofloxacin (500 and 750 mg, respectively) as well as oral oseltamivir (75 mg) twice daily for 5 days were added to the initial treatment.

Despite these prescriptions (on the 7th day of clinical evolution), the patient showed no clinical improvement and was attended at Culiacan General Hospital, where she presented slight pallor, respiratory rate of 22–24 breaths per minute, and bilateral pulmonary crackles, but with an oxygen saturation of 97 % and neurological integrity.

Abnormal laboratory tests included leukocytosis, neutrophilia, lymphocytosis, and elevated C-reactive protein. Both influenza and COVID-19 tests were negative, whereas computed tomography of the chest showed bilateral consolidations, so unspecified viral pneumonia was suspected. Accordingly, injectable acetaminophen (1 gr), nebulizations with ipratropium/salbutamol and budesonide (0.5 mg/2.5 mg/250 mg), intravenous and oral levofloxacin (500 and 750 mg, respectively), intramuscular dexamethasone (8 mg), and oral oseltamivir (75 mg) daily for 6 days were prescribed. Due to alleged clinical improvement, the patient was discharged after 24 hours of hospital observation.

On the 14th day of clinical evolution, the patient was readmitted to the hospital because of generalized pallor, rhinorrhea, worsening cough, blood pressure of 98/55 mmHg, temperature of 35.6 °C, and bilateral pulmonary crackles, but normal respiratory and heartbeat rates (18 and 86 per minute, respectively). Additional physical examination was uneventful, while leukocytosis, neutrophilia lymphocytosis, and elevated C-reactive protein were noted. Owing to the previous therapeutic approach, unspecified pneumonia was updated as a presumptive diagnosis, and meropenem antibiotic was included (dexamethasone was removed). In the following 24 hours, her blood pressure was 134/89 mmHg, and chest X-ray depicted multiple bilateral infiltrates, suspecting pulmonary tuberculosis; but indian ink, KOH preparation, acid-fast bacillus, and urocultive microbiologic tests were negative.

On the 17th day of clinical evolution, the patient was admitted to the intermediate care unit as a result of respiratory distress syndrome reaching a Richmond agitation sedation scale −5 (unarousable). Orotracheal intubation was carried out, and vital signs were as follows: heart rate 113 beats per minute, blood pressure 99/55 mmHg, temperature 36.2 °C, and oxygen saturation 74 %. Then, clindamycin (600 mg/day) and linezolid (600 mg/12 hours) to prevent methicillin-resistant *Staphylococcus aureus* infection were administered. Clinical follow-up showed persistent systemic decay of health characterized by hypotension, anuria, bradycardia, peripheral cyanosis, and oxygen saturation of 70 %. Also, severe mixed acidosis and acute kidney injury (scale 3 of kidney disease improving global outcomes) were observed. Altogether, severe acute respiratory distress syndrome with refractory septic shock of pulmonary origin and severe necrotizing pneumonia of undetermined etiology was determined. The patient went into refractory cardiac arrest, and death was registered on the 19th day of clinical evolution.

**Fig. 1:**
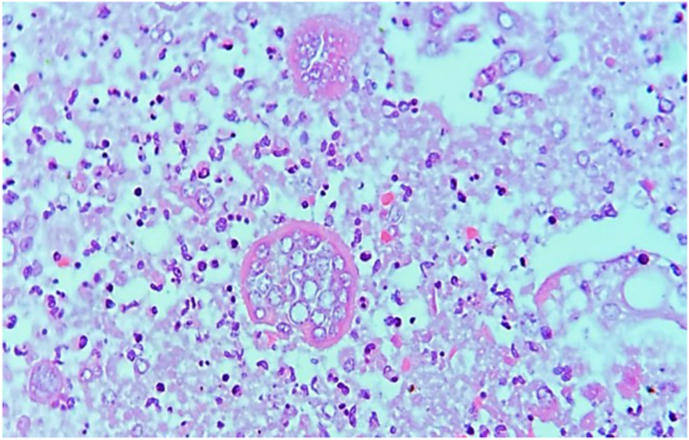
Hematoxylin and eosin-stained histological section (40× magnification) with necrosis of the pulmonary tissue and spherules.

**Fig. 2:**
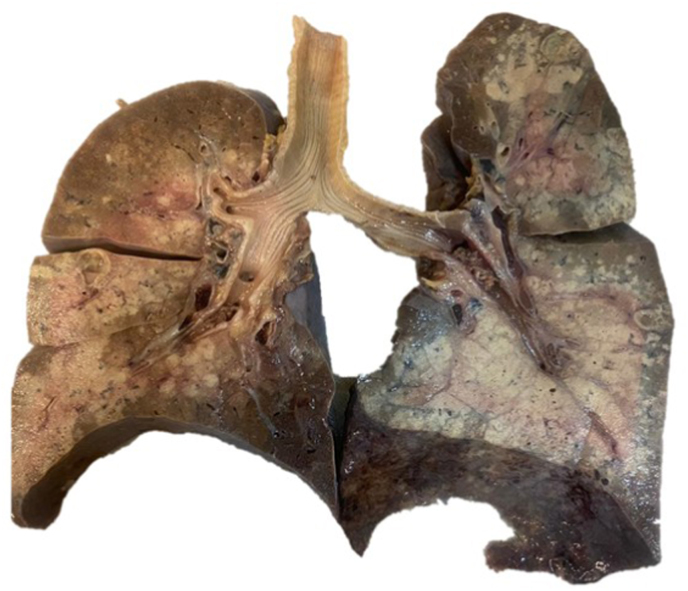
Vertical section of both lungs showing extensive areas of congestion and hemorrhagic necrosis.

**Fig. 3:**
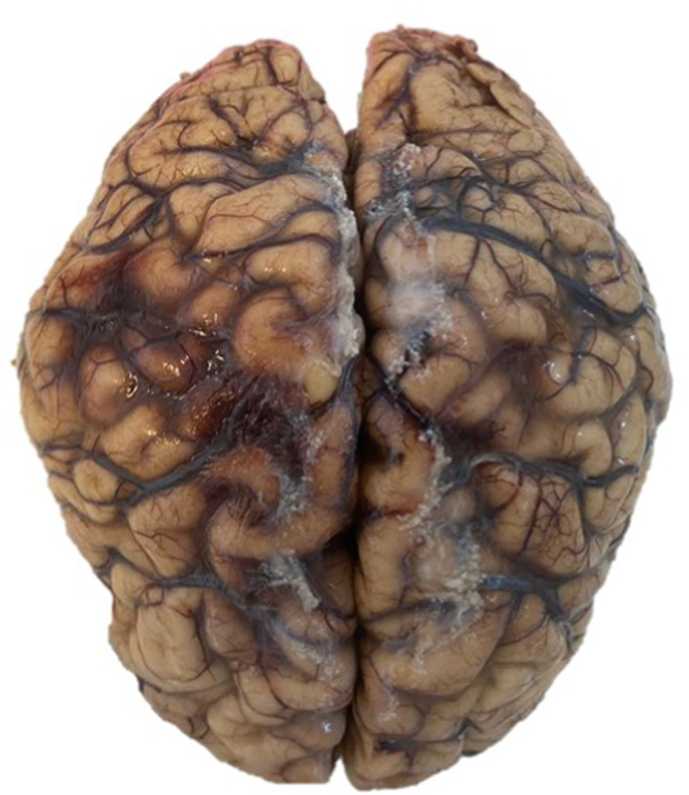
Top view of the brain with hemorrhage and vascular congestion of the arachnoid with signs of cerebral edema.

**Fig. 4:**
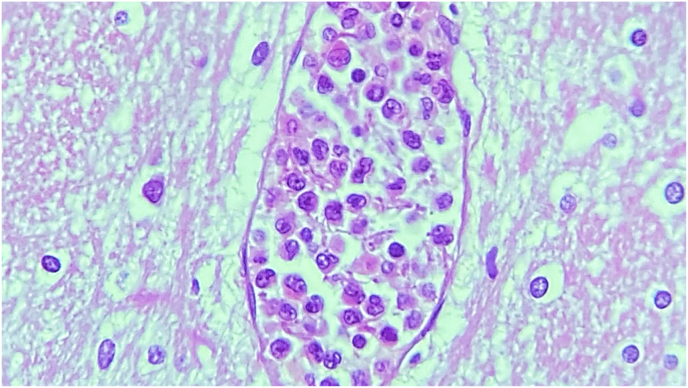
Histological section of brain tissue showing edema and a blood vessel with abundant inflammatory cells (40× magnification).

Next of kin relative granted the patient's autopsy. Microscopic examination revealed multiple spherules in both lungs (Fig. 1) and lymph nodes with chronic inflammation consistent with bilateral pulmonary coccidioidomycosis and necrosis (Fig. 2). Macroscopic appraise disclosed nodular pattern lesions across the lungs, liver, spleen, kidneys, and brain tissues. Additional exploration showed diffuse alveolar damage, lymphoid depletion, vacuolation of myocardial fibers, ischemic injury of the upper gastrointestinal, pancreatic, hepatic, splenic, bilateral renal, and suprarenal tracts, multivisceral hypoxic-ischemic myopathy, hypoxic-ischemic encephalopathy, acute meningitis (Fig. 3), multivisceral vascular congestion, fat necrosis associated with chronic pancreatitis, and chronic active hepatitis. The histologic cuts of brain tissue showed edema and a blood vessel with abundant inflammatory cells, without spherules (Fig. 4).

## 3 Discussion

This report describes an immunocompetent young woman, without evident risk factors, who faced a progressive and lethal outcome as a result of pulmonary coccidioidomycosis. Mortality-related coccidioidomycosis involves particular clinical characteristics, as disease progression is usually fatal in immunosuppressed patients and disseminated forms [4,5], and contrast to this report. Well-known risk factors may also contribute to coccidioidomycosis, such as occupational soil exposure, age ≥55 years, pregnancy, or comorbidities [6]; yet, all these determinants were absent in the reported patient.

On one hand, it is surmised that the patient's *Coccidioides spp*. exposure could be related to conversation and other interactions with the passengers neighbouring as a taxi driver; however an autochthonous spread could take place since Northwest Mexico is deemed endemic for coccidioidomycosis [7]. It must be noted that this geographical setting has the highest rates of coccidioidin immunoreaction [8], and cases with notable clinical variability have been reported [[9], [10], [11]]. On the other hand, the aberrant clinical progression and subsequent death in an immunocompetent state without potential risk factors is extremely infrequent [12], and does not follow the expected clinical course. Receiving corticosteroids may be a plausible explanation for the patient's outcome, because it is considered a common risk factor for the extrapulmonary form [1]. Further investigations are needed since supporting evidence mainly proceeds from retrospective observations.

In this context, in a cohort of patients treated with corticosteroids due to autoimmune illness, a total of 107 cases were diagnosed with coccidioidomycosis. Measures considering the relative risk for coccidioidomycosis revealed an adjusted hazard ratio of 2.29 compared to those without receiving corticosteroids [13]. Furthermore, experimental data from an ortholog model (B6D2F1 mice) infected with *Coccidioides posadasii* indicated a lethal outcome around days 16-25 associated with dexamethasone-induced immunosuppression [14].

The seemingly poor adverse outcomes due to short-term corticosteroid therapy in coccidioidomycosis have also been documented [15,16]. These discrepancies might shed light on the present patient's clinical course and demise, as therapeutic interactions and adverse effects of corticosteroids and antibiotics can contribute towards an impaired immunity response in the host [15,17].

In line with the above mentioned, receiving antibiotics can also influence the lung microbiota by promoting respiratory dysbiosis and may have pathogenic implications for the host's immune system [18]. This particular interaction seems to be a predictor of mortality in mechanically ventilated patients [19], and concurs with this report. Moreover, gene mutations in *LEC7A* and *PLCG2* have been associated with disseminated coccidioidomycosis and may modify the pleiotropic effects of tumor necrosis factor-alpha in the immunocompetent host, counteracting *Coccidioides spp.* [3].

The impact of these genes in relation to the patient's clinical expression was not assessed, but it is improbable as her medical background lacks any history of autoimmune illness or recurrent infections, as would be expected in these mutations.

Taken together, myriad clinical factors should be considered in patients who undergo mortality-related coccidioidomycosis. For example, pregnancy, chronic kidney disease, and acquired immunodeficiency syndrome were the most concurrent factors among 31 cases reported at a referral hospital in Northeastern Mexico [20]. Of note, there were three deaths lacking any associated factors for *Coccidioides* infection susceptibility. Other patients have experienced chronic neurological-related coccidioidomycosis symptomatology in an immunocompetent state [21], or may occur in non-endemic geographical settings [7].

Finally, mortality-related coccidioidomycosis along with an immunocompetent state among the pediatric population may befall, as in a Mexican cohort of 30 pediatric cases, two deceases without evidence of known risk factors, were reported [22].

## 4 Conclusion

Within this framework, findings in this report highlight controversial but useful clinical contributions regarding mortality-related coccidioidomycosis in immunocompetent patients. The latter may be singularly advantageous in limited clinical settings, as clinical diagnosis of *Coccidioides* infection may be challenging. Given that symptoms overlap with a respiratory infection and are typically mild or subclinical in immunocompetent patients, intensive monitoring and specific treatment in endemic regions are recommended so that eventual mortality-related coccidioidomycosis can be prevented.

## CRediT authorship contribution statement

**Efrén Rafael Ríos-Burgueño:** Validation, Supervision, Methodology, Investigation, Conceptualization. **Luis Antonio Ochoa-Ramírez:** Methodology, Investigation. **Ismael Velarde-Rodríguez:** Supervision, Investigation, Data curation. **Juan Manuel Ramírez-Sánchez:** Writing – review & editing, Writing – original draft, Data curation. **Víctor Michael Salinas-Torres:** Writing – original draft, Methodology, Data curation. **Jesús Salvador Velarde-Félix:** Writing – original draft, Supervision, Methodology, Investigation, Conceptualization.

## Funding

We had no financial support for this study.

## Ethical approval and informed consent

We have obtained informed consent of the patients's mother.
